# Atypical Erythema Migrans in Patients with PCR-Positive Lyme Disease

**DOI:** 10.3201/eid1905.120796

**Published:** 2013-05

**Authors:** Steven E. Schutzer, Bernard W. Berger, James G. Krueger, Mark W. Eshoo, David J. Ecker, John N. Aucott

**Affiliations:** University of Medicine and Dentistry of New Jersey–New Jersey Medical School, Newark, New Jersey, USA (S.E. Schutzer);; Private Dermatology Practice, Southampton, New York, USA (B.W. Berger);; Rockefeller University, New York, New York, USA (J.G. Krueger);; Ibis Biosciences Inc., Carlsbad, California, USA (M.W. Eshoo, D.J. Ecker);; The Johns Hopkins School of Medicine, Baltimore, Maryland, USA (J.N. Aucott)

**Keywords:** erythema migrans, rash, bull’s-eye rash, Lyme disease, Borrelia burgdorferi, bacteria, PCR

**To the Editor:** The best diagnostic sign in patients with early Lyme disease is a skin lesion, erythema migrans (EM). However this sign may not occur or be recognized in 30% of cases (*1*). Furthermore, the EM rash may not display a classic bull’s-eye (ring-within-a-ring) appearance, a fact that may be underappreciated (*2**,**3*). Some studies noted uncharacteristic variants of EM in 25%–30% of cases (*4*–*7*). One study reported the rash to be uniformly red in 60% of cases (*6*). Other atypical variants of EM are a blue-red appearance and, occasionally, a vesicular central region (*4**,**5*). We describe the occurrence of atypical EM in patients with microbiologically proven *Borrelia burgdorferi* infection.

During spring and summer 2009, a total of 29 patients with classic or possible EM and suspected Lyme disease were referred by primary care physicians for an ongoing prospective study. Laboratory methods have been described (*8*). The patients were >18 years of age and lived in suburban Baltimore, Maryland, USA, where Lyme disease is endemic. All patients had extracutaneous manifestations (e.g., virus-like symptoms). Fourteen patients met laboratory criteria for study analysis: 1) positive PCR at the initial study visit, detected by a *B. burgdorferi*–specific nucleic acid–enhancing PCR method on a 1.25-mL whole blood sample (*8*), and 2) evidence of *B. burgdorferi* exposure by the 2-tiered antibody test at the initial or posttreatment visit. Other entry criteria were a rash >5 cm and symptoms compatible with early Lyme disease (*1*); exclusion criteria were certain preexisting medical conditions (*8*).

A panel of experienced specialists, including dermatologists, were shown photographs of the patients’ skin lesions and asked if they would expect the average primary care physician to diagnose the lesions as EM. To avoid bias, PCR and serologic test results we withheld from the specialists and they were asked to categorize lesions by characteristics common to target-like and non–target-like lesions. Lesions with the classic bull’s-eye appearance, with central clearing and peripheral erythema, were classified as classic EM; those with uniform red or red-blue or other appearance and lacking central clearing were classified as possible atypical EM. If any lesion of a multiple lesion set was classic in appearance, we categorized the rash as classic EM. Of the 14 patients with positive PCR, 10 had nonclassic EM (Figure) and 4 had classic, target-like EM. Atypical rashes varied from those close to classic EM to those resembling lesions more common in other conditions (e.g., insect or spider bites) and, consequently, prone to misdiagnosis.

**Figure:**
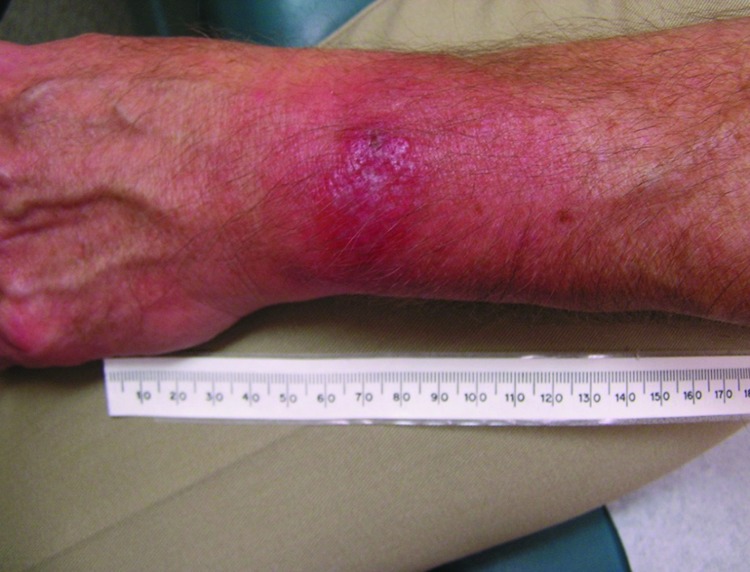
Atypical erythema migrans lesion on a patient with PCR-positive result for *Borrelia* *burgdorferi* infection. The rash was not considered typical because it lacked central clearing and peripheral erythema. The differential diagnosis included a contact dermatitis and arthropod bite. At the initial examination, this patient was seronegative for *B. burgdorferi* by 2-tiered criteria. Three weeks after therapy, the patient had positive results for ELISA and IgM Western blot and negative results for IgG Western blot, providing evidence of seroevolution (i.e., increasing antibody titer and/or increase in band intensity or appearance of new antigen bands to *B. burgdorferi*).

Depending on the appearance of an atypical rash, the differential diagnosis could include contact dermatitis, arthropod bite, or, in cases with annular lesions, fixed drug eruptions, granuloma annulare, cellulitis, dermatophytosis, or systemic lupus erythematosus (*5*). In addition, a diagnosis can be more challenging when there are multiple skin lesions rather than a single lesion and in a pattern unfamiliar to a general practitioner.

Multiple textbooks and websites have featured pictures of EM as a bull’s-eye lesion (Technical Appendix). This emphasis on target-like lesions may have inadvertently contributed to an underappreciation for atypical skin lesions caused by Lyme disease. Nevertheless, physician recognition of Lyme disease–associated EM is essential because current approved laboratory tests may not identify *B. burgdorferi* in the first few weeks of infection (*8*), when an accurate diagnosis can lead to early curative therapy.

Separate studies found different percentages of atypical Lyme disease–associated rashes (*3**,**4**,**9*); each was lower than the percentage found in our study. Our study has several limitations: it encompassed only 1 recruitment season, 1 geographic site, and a small number of patients. The sensitivity of PCR for blood specimens is improving (*8*); however, PCR may have missed some acute cases in our study for reasons cited below. Therefore, these patients should not obligatorily be considered as representative of all acute Lyme disease patients.

Our study results serve as an impetus for studying more patients with systemic and nonsystemic signs and symptoms over multiple seasons and geographic areas and for including PCR analysis of skin lesions in future studies. PCR of skin biopsy samples may provide insight as to whether a negative blood PCR is the result of infection with a skin-restricted strain (*10*) in patients in whom bacterial dissemination is not expected or a result of low copy number of *B. burgdorferi* in the blood sample.

Our results serve as a reminder that patients with early Lyme disease may have an atypical rash, not the classic (textbook) bull’s-eye lesion. Close observation and a detailed history of whether the rash is enlarging, has enlarged, or is spreading should be part of the consideration of the diagnosis. Observation for extracutaneous signs of early infection, such as cranial seventh nerve palsy (Bell’s palsy) or meningitis, is also essential.

In summary, the EM rash of Lyme disease can have an atypical appearance. Thus, clinicians should consider Lyme disease in the differential diagnosis of patients who have a rash that may not be classic EM and who have been in areas where Lyme disease occurs.

Technical Appendix Classic Lyme disease erythema migrans rash.
