# Supplementary material for: Atypical Erythema Migrans in Patients with PCR-Positive Lyme Disease
Source: Emerg Infect Dis. 2013 May;19(5):815–7. doi: 10.3201/eid1905.120796 (PMC3647494; doi:10.3201/eid1905.120796)
Supplement: Technical Appendix — Classic Lyme disease erythema migrans rash. [file 12-0796-Techapp-s1.pdf]

Article DOI: http://dx.doi.org/10.3201/eid1905.120796

Atypical Erythema Migrans in Patients with PCR-Positive Lyme Disease

Technical Appendix

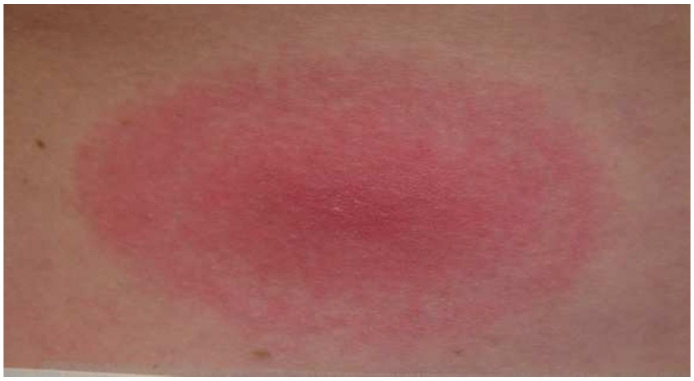

Figure. Classic bull's-eye (ring-within-a-ring) erythema migrans lesion (5 cm). Whole blood PCRs of samples obtained at the patient's initial medical visit were positive for *Borrelia burgdorferi*, and results of 2-tiered serologic testing were negative. The patient seroconverted after a 3-week course of antimicrobial drug treatment.
